# Computed tomographic and radiographic examination of dental structures in South American camelid specimen of different ages

**DOI:** 10.1186/1746-6148-10-4

**Published:** 2014-01-06

**Authors:** Abdolrazagh Rostami, Urs Geissbühler, Frank Schellenberger, Patrik Zanolari

**Affiliations:** 1 Clinical Radiology, Department of Clinical Veterinary Medicine, Vetsuisse-Faculty, University of Berne, PO Box 8466, Berne 3001, Switzerland; 2 Department of Surgery and Radiology, Faculty of Veterinary Medicine, University of Tehran, PO Box 1419963111, Tehran, Iran; 3 Frank Schellenberger, Sandweg 5, Waldkirch 79183, Germany; 4 Clinic for Ruminants, Vetsuisse-Faculty, University of Berne, PO Box 8466, Bremgartenstrasse 109a, Berne 3001, Switzerland

**Keywords:** Llama, Alpaca, Teeth, Radiography, CT

## Abstract

**Background:**

Tooth root problems and periodontal diseases are common in South American camelids (SAC). The objective was to evaluate and optimize the imaging technique for dental radiography in SAC and to describe the radiographic and computed tomographic (CT) anatomy of normal teeth at different ages. In this study, the heads of 20 healthy SAC slaughtered for meat production or euthanized for reasons not related to dental problems included 7 female and 10 male llamas and 3 male alpacas. Using a standardized protocol, radiographs and CT scans of the 20 specimen were performed.

**Results:**

The most useful radiographic projections for mandibular and maxillary cheek teeth evaluation turned out to be lateral30°ventral - laterodorsal and lateral30°dorsal - lateroventral with slight separation of the dental arcades respectively. Digital radiographic and CT appearance of the mandibular and maxillary teeth were described from the beginning of mineralization till maturity. In addition the normal range of the CT radio density of different cheek teeth and different dental tissues were measured. Hounsfield units of different dental tissues of SAC turned out to be similar to equids.

Deviation, shortening and partial destruction of the distal tooth root of mandibular 09′s and 10′s and of maxillary 09′s was observed and the existence of a common pulp chamber in younger teeth was revealed.

**Conclusions:**

The present study provides information about the dental imaging morphology in clinically healthy SAC. This basic information provides fundamental knowledge for evaluating images and planning treatments in clinically affected animals.

## Background

Root problems and periodontal diseases are common in South American camelids (SAC) [1-3]. Malocclusion [3,4], periodontal abscess, infection of the cheek teeth [2,3,5,6], alveolar periosteitis or osteitis [3], and oral neoplasia [7] are various manifestations of dental problems in SAC of all ages. As in other species, radiography is a useful method of providing diagnostic information [8].

Computed tomographic (CT) images of the skull are superior to radiographs due to the higher contrast resolution, the lack of superimposition and the possibility of multiplanar and three dimensional (3D) reconstructions. Therefore, CT images may reveal bone and tooth abnormalities earlier than radiography. Diagnoses based on CT imaging allow a more accurate prognosis and therapeutic plan [9].

Despite the common occurrence of dental diseases and the routine use of radiography [1,5,6,9], little information has been published about diagnostic imaging of the normal dental arcades in SAC.

The purpose of this study was to evaluate and optimize the imaging technique for dental radiography in SAC and to describe the radiographic and CT anatomy of normal teeth at different ages.

## Methods

### Specimen

The heads of 20 healthy SAC slaughtered for meat production or euthanized for other reasons not related to dental problems were collected. The study population consisted of 7 female and 10 male llamas and 3 male alpacas (Table 1). The median age of the animals was 3 years and 3 months ranging from 9 months to 9 years. None of them had a history of disease or abnormality in the area of the head. Each head was separated from the neck at the level of the atlanto-axial joint, and frozen at minus 20°C until imaging evaluation. For radiographic and CT examination, the heads were thawed three days in advance.

**Table 1:** Used specimens related to their species, gender and age

**Number**	**Species**	**Sex**	**Age months**
1	Llama	Female	36
2	Llama	Male	9
3	Llama	Female	18
4	Llama	Female	39
5	Llama	Female	107
6	Alpaca	Male	67
7	Llama	Male	41
8	Llama	Male	80
9	Llama	Male	61
10	Llama	Female	44
11	Alpaca	Male	9
12	Alpaca	Male	61
13	Llama	Male	38
14	Llama	Female	31
15	Llama	Male	32
16	Llama	Female	19
17	Llama	Male	32
18	Llama	Male	32
19	Llama	Male	30
20	Llama	Male	21

The modified Triadan system was applied providing a consistent method of numbering the teeth (Table 2) [10,11].

**Table 2:** Modified Triadan numbering system

**Old nomenclature**	**I1**	**I2**	**I3**	**C**	**PM1**	**PM2**	**PM3**	**PM4**	**M1**	**M2**	**M3**
Modified Triadan	01	02	03	04	05	06	07	08	09	10	11

The modified Triadan numbering system consists of 3 numbers. The first number relates to the quadrant of the mouth in which the tooth lies; Upper Right: 1; Upper Left: 2; Lower Left: 3; Lower Right: 4.

If a deciduous tooth is referred to, then a different number is used: Upper Right: 5; Upper Left: 6; Lower Left: 7; Lower Right: 8. The second and third numbers refer to the location in rostrocaudal orientation.

### Diagnostic imaging technique and image evaluation

**Figure 1:**
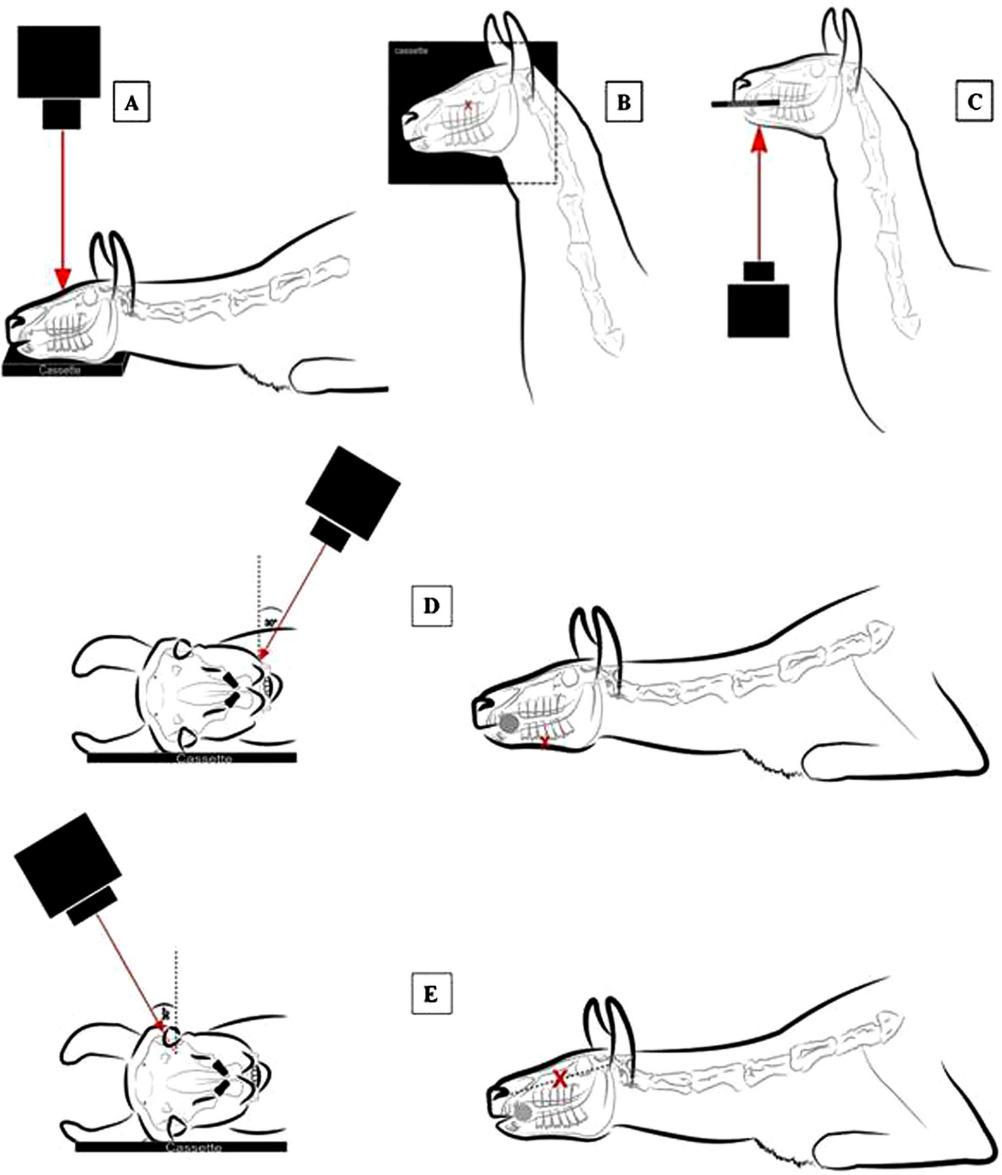
**Radiographic projections. A**: Dorsoventral projection. **B**: Laterolateral projection, x = central beam. **C**: Intraoral ventrodorsal projection. **D**: Left to right lateral30°ventral - laterodorsal oblique projection, x = central beam. **E**: Left to right lateral30°dorsal - lateroventral oblique projection, x = central beam.

First, CT scans were performed using a standardized protocol. The specimens were positioned with the hard palate parallel to the CT- table. Open- and closed-mouth transverse images were obtained of each head using a 16-slice CT scanner^a^. Dental algorhythms were used for image acquisition. Exposure data were 120 kVp and 200 mAs, and a pitch of 0.563 was used. Images of 1 mm slice thickness were acquired. The window width and the window level were set at 2000 and 800 Hounsfield units, respectively. Multiplanar reconstruction (MPR) and 3D reformatted images were calculated in order to compare the radiographic and CT findings and to evaluate the appropriate radiographic projections. Five different radiographic projections were performed to completely examine the teeth arcades of the upper and lower jaw (Table 3, Figure 1). Using a standardized protocol digital radiographs were obtained with high frequency stationary x-ray equipment and a computed radiography system^b,c^. Exposure factors for head overview were applied to produce dental projections [Focal film distance (FFD) = 60 cm, voltage = 60 kV and product of amperage and exposure time = 20 mAs]. Images were acquired without a grid. An imaging plate^d^ with a size of 35.5 × 43 cm was used for all projections, except for intraoral ventrodorsal (VD) projections where a mammography plate^e^ with a size of 18 × 24 cm was used. The heads were positioned on the imaging plate. For the intraoral projection, the heads were fixed in dorsal recumbency and the plate was inserted into the mouth to the level of the caudal edge of the diastema. Pieces of foam rubber were used to position the heads on the table. For lateral and oblique projections the imaging plate was put in close contact to the surface of the heads in a vertical orientation by means of a cassette holder. For oblique projections the heads were fixed on top of two upright stands in sternal recumbency. The in-built goniometer of the radiology machine was used to produce the intended incident angle calculated in CT. In laterolateral and lateral oblique open-mouth projections, a radiolucent 20 ml syringe with an outer diameter of 21 mm was put horizontally between upper and lower jaws, caudal to the canine teeth to keep dental arches separated and to avoid superimposition of maxillary and mandibular dental arcades. Dorsoventral and intraoral projections were produced without a gag.

**Table 3:** Radiographic positioning

**Projection**	**Description of the projections**	**Central beam**
**Dorsoventral**	Both mandibles positioned on the plate, hard palate parallel to the table, vertical beam	Cross point of midline and line between two MOCs*
**Laterolateral**	Sagittal plane of the head parallel to the plate, horizontal beam	Half distance between ear base and lateral nostril angle on the facial crest
**Left to right lateral30°dorsal-lateroventral oblique**	Imaging plate parallel to the sagittal plane of the head	Half distance between ear base and lateral nostril angle on the line parallel to nasal margin which goes through lateral nostril angle
**Left to right lateral30°ventral-laterodorsal oblique**	Imaging plate parallel to the sagittal plane of the head	Half distance between ear base and lateral nostril angle on the rim of the corpus mandibulae
**Intraoral VD of lower jaw**	Intraoral plate, vertical beam	In the midline at the caudal aspect of the symphysis mandibulae

Radiographic positioning technique for complete survey of dental arcades (*MOC: medial ocular canthus).

**Figure 2:**
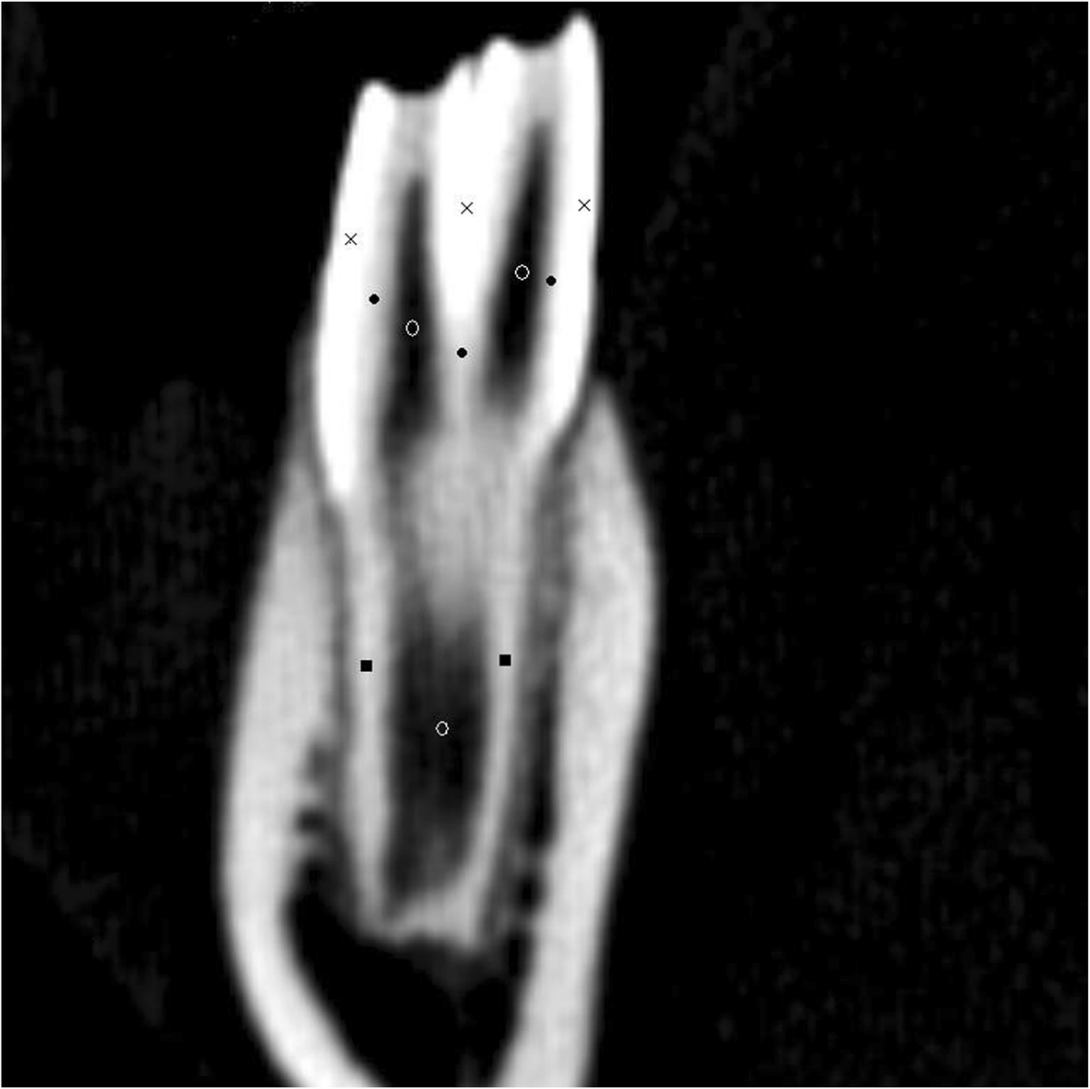
**Dental areas where the radio density of different tissues were measured.** As indicated below, for each tissue two or three locations of the tooth were selected and the Hounsfield units (HU) were measured as the average of the calculated HU of these locations. The same criteria were applied to all teeth. ×: enamel, •: dentine, ○: pulp, ■: cementum.

All images were sent to a picture archiving and communication system (PACS), reviewed with film-reading software^f^, and evaluated to describe the radiographic anatomy and relationship of different teeth to each other. Hounsfield units of different dental areas were measured in the film-reading software. For each dental tissue two or three locations were measured and the average value for each tissue was reported (Figure 2).

Statistical analysis was performed by use of commercially available software^g^. For all measurements, the mean and standard deviations were calculated.

## Results

### Incisors and canines

The mandibular incisors were best visualized in the intraoral VD projection. The third incisor (03) of each hemi-mandible was subjectively narrower than the first (01). The deciduous incisors of both, alpacas and llamas were spatula-shaped. The permanent incisors of the llamas were still spatula-shaped with tapered roots, whereas the permanent incisors of alpacas were rectangular in cross section and chisel-shaped.

**Figure 3:**
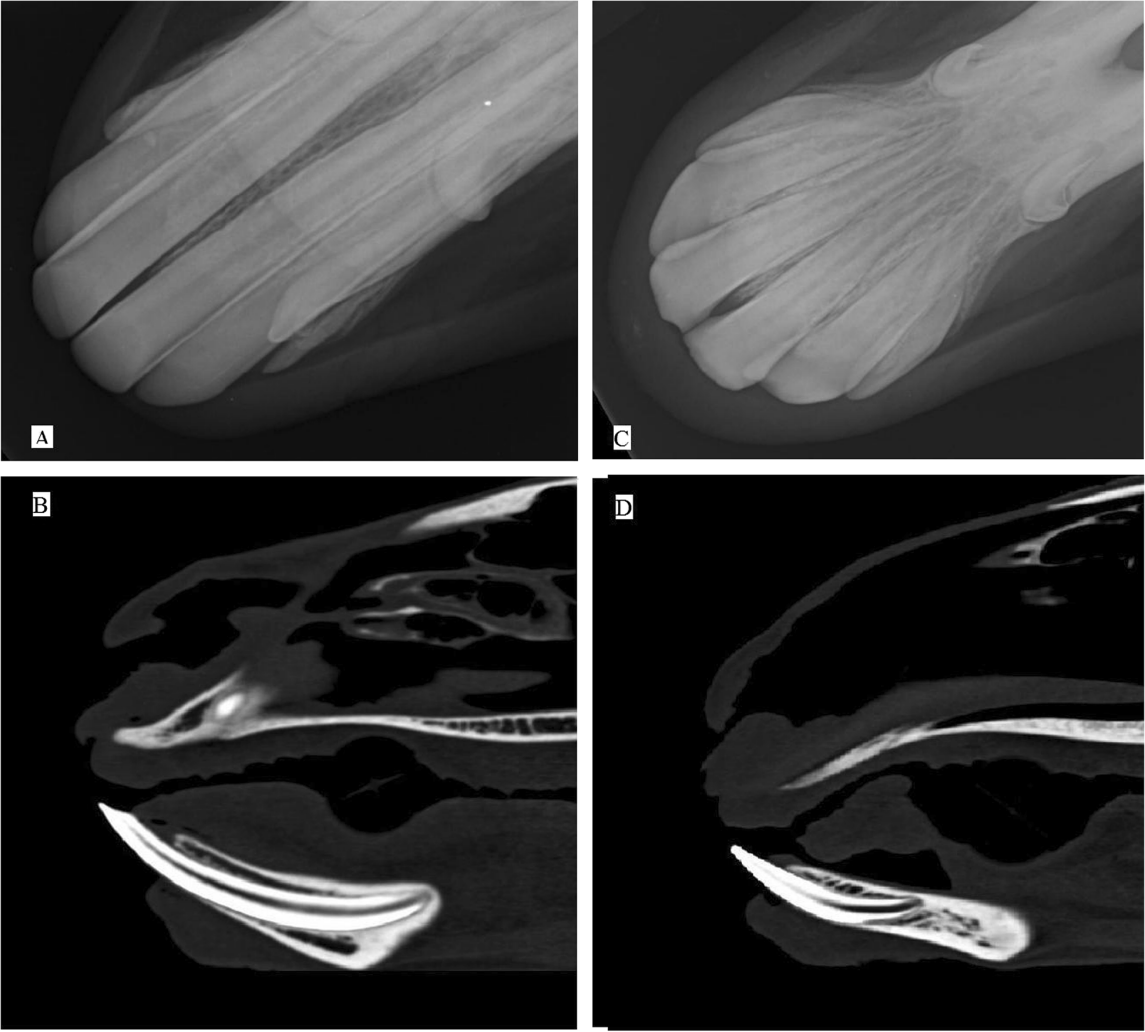
**Mandibular incisors and canine teeth.** Intraoral VD projection of the lower jaw of a 5 years old male alpaca **(A)** and a 4 years old female llama **(C)**. Chisel-shaped permanent incisors of alpacas and spatula-shaped permanent incisors of llamas with their tapered roots are best visualized in this view. Note the canine teeth of both species are curved. Sagittal para-median MPR reconstructed CT images of the same animals in distal row **(B and D)** show the incisors anchored in alveolar bone. Sharp tip and apex of the incisors is obviously visualized.

The mandibular canine tooth of both species had a curved shape, was round in cross section and was surrounded entirely by enamel at the erupted and intraalveolar crown. The tooth was sharply pointed at both coronal and apical ends and anchored in the alveolar bone by a curved root (Figure 3).

There was one incisor (03) and one canine tooth (04) in the maxillary arcade of each side. These two teeth were curved and sharp at both ends with a round cross-sectional structure. The crowns were completely covered with enamel.

### Dental development and morphology of cheek teeth

South American camelids had cheek teeth with a typical hypsodontal structure. Their enamel folds made typical palisade-like segments, here called columns, in both the crown and reserve crown region. These columns had one rostrocaudal pulp horn segment, which, together with infundibular invagination, created a typical selenodont occlusal surface.

**Figure 4:**
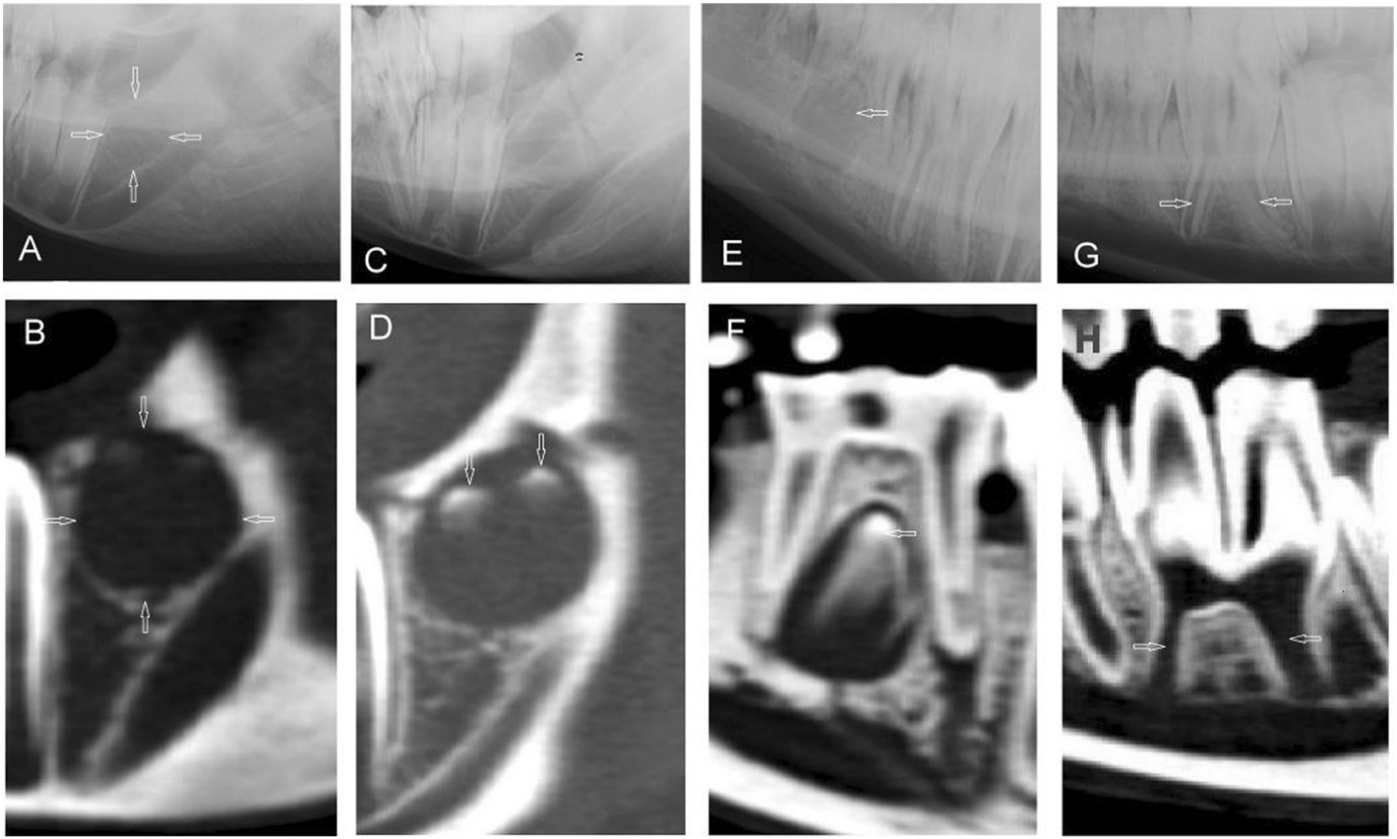
**Different stages of the development of the dental sac.** Lateral30°ventral - laterodorsal radiographs (upper row) and corresponding sagittal CT-reconstructions (lower row) of the development of the dental sac in different stages. **A**, **B**: Early radiolucent dental sac for mandibular 10′s in a 9 month old alpaca. **C**, **D**: Coronal mineralization of the mandibular 10 with two enamel columns in a 9 month old llama (not visible in C). **E**, **F**: Erupting mandibular permanent 08 in a 30 month old llama, which would cause the deciduous tooth shedding down later. **G**, **H**: Wide pulp cavities and a common pulp chamber with open apical foramina in a recently erupted mandibular 08 in a young llama (age 32 months). Described findings are indicated with white arrows.

At the beginning of dental development, a radiolucent, well demarcated area appeared at the location of a newly forming tooth (Figures 4A, 4B). In each sac, the number of mineralization centers in the sagittal CT reconstruction showed the number of dental columns of the newly erupting tooth. The process of mineralization started from the occlusal part (Figures 4C, 4D) and continued to the apical part of the different teeth columns. With age, a pulp cavity and a wide open apical foramen appeared in each column. The mineralization narrowed the apical foramina and the pulp cavities.

Each separate molar column contained two pulp horns, except the distal column of the third mandibular molar (11). Here, the pulp was not divided and had only one horn. All molars had 4 pulp horns and mandibular 11′s had 5 pulp horns. These pulp horns tended to be narrower toward the occlusal surface. In younger teeth of SAC a common pulp chamber was clearly visible. During the aging process of the tooth, secondary dentine is built up. This reduces the overall pulp cavity size and closes the common pulp chamber. Interpulpar communications between the pulp horns of the respective column were not detected with our CT-examination technique. The pulp cavities had soft tissue opacity.

The enamel folded around the pulp horns and was invaginated between the two pulp horns of the same column, producing an infundibulum in every molar column except for the distal column of mandibular 11′s which had no infundibulum. So there were two infundibula in each molar. In cross section, these infundibula had a crescent shape of which the convex aspect in mandibular teeth was located buccally and in maxillary teeth palatinally. The infundibula were filled with cementum and stretched to the occlusal surface of the teeth. No signs of infundibular lesions, but occlusal exposure and filling of infundibula with food debris were detected with age.

### Mandibular cheek teeth

**Figure 5:**
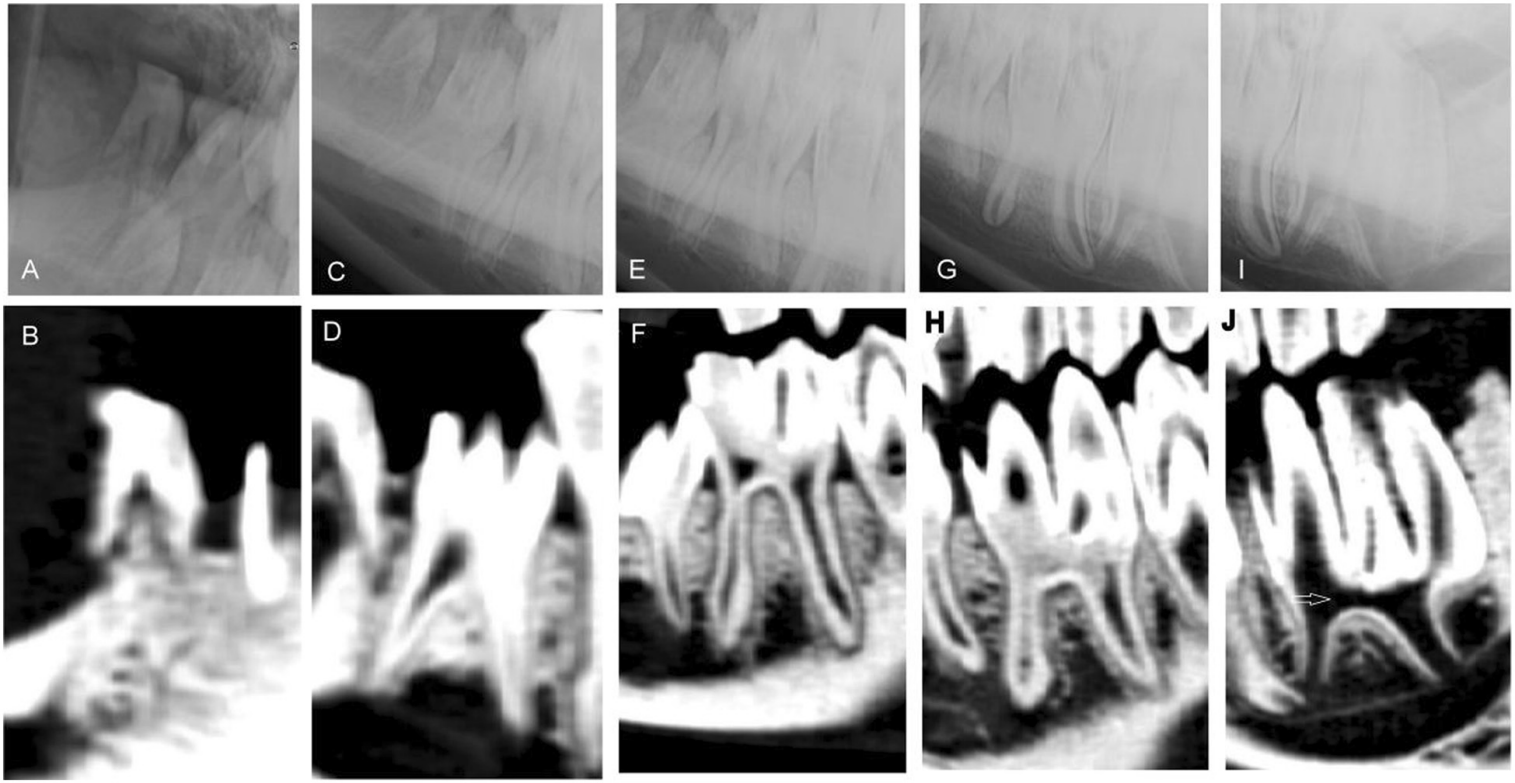
**Mandibular cheek teeth.** Mandibular cheek teeth of an adult llama (age 44 months). There were two premolars and three molars on each hemi-mandible. A/C/E/G/I: Left to right lateral30°ventral-laterodorsal oblique projection showing the right mandible. B/D/F/H/J: Sagittal CT-reconstructions of mandibular 07 to 11. **A** and **B**: 07, **C** and **D**: 08, **E** and **F**: 09, **G** and **H**: 10, **I** and **J**: 11. J shows a stage, where the common pulp chamber is still existing (white arrow).

The mandibular 07 was present in 13 (65%) cases and had one conical column in lateral oblique projection and two close roots (Figures 5A, 5B). The 07 was the smallest mandibular cheek tooth. Both deciduous and permanent 07′s were similar in shape but the permanent teeth were larger.

The mandibular 08 was present in all specimens. The mandibular deciduous 08 had three columns and just two divergent roots. The two mesial columns shared one root. The mandibular permanent 08 had two columns and two roots and was triangular in shape. The mandibular premolars had no infundibulum (Figures 5A, 5B).

The mandibular 09 and 10 were made up of two separate columns which were attached together in crown and each was anchored in alveolar bone with a separate single root (Figures 5A, 5B). The 10′s were larger than 09′s (Figures 5C, 5D).

The columns of 09, 10 and two mesial columns of 11 had a rectangular shape in cross section, with a dome shaped cap attached to their buccal aspect. In the cross sectional images, the entire mandibular dental arcade formed a straight line with enamel invaginations between the columns in the lingual aspect and a palisade like surface with vaults in the buccal aspect. The mesial columns of 10′s and 11′s had an additional enamel fold which was tightly attached to the mesio-buccal border of the teeth and was more pronounced in 11′s. In lateral projection the occlusal surface of the teeth was serrated and had sharp enamel crests. No interdental space was present at the level of the occlusal surface of the cheek teeth and they were in tight contact, making one entire grinding surface in each arcade.

The 11′s had three columns and each column had its own single root (Figures 5I, 5J). These roots were apparently shorter than the roots of other cheek teeth in comparison to their crown (Figure 5). The third column of 11′s, located at the distal aspect, was smaller than the other columns and had an oval shape in cross section. It has no infundibulum and only one pulp horn The roots and columns were located side by side in the same plane.

**Figure 6:**
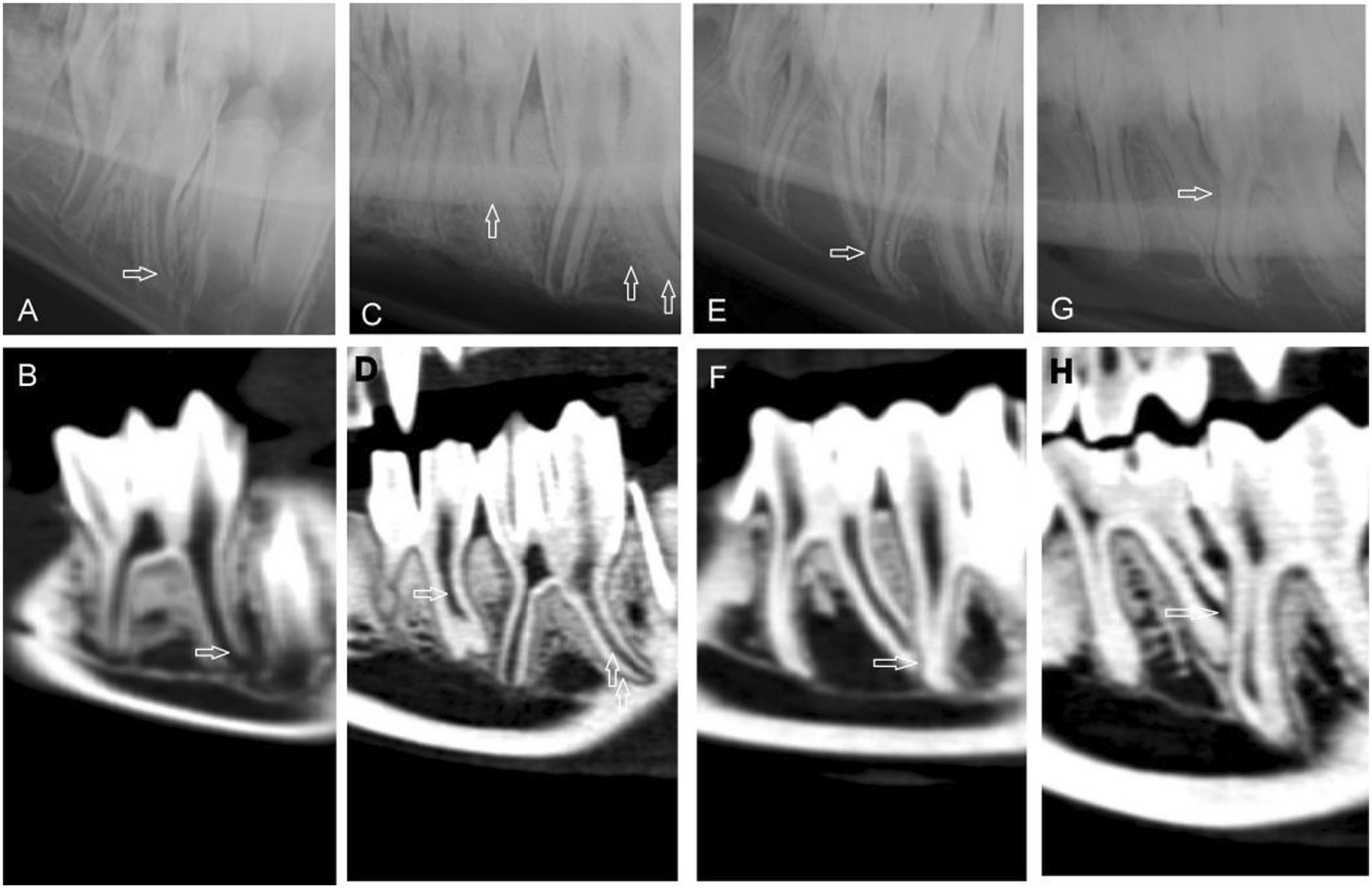
**Findings in curved mandibular tooth roots at different ages.** Digital radiographs (lateral30°ventral - laterodorsal oblique projection, upper row) and sagittal CT-reconstructions (lower row) of mandibular teeth showing distal root shape in different ages. **A**/**B**: Caudal deviation of the distal root of a deciduous 08 in a 9 month old alpaca. **C**/**D**: Sclerotic changes in the alveolar bone and curved shape of the distal roots of 409 and 410 in a 31 month old llama. **E**/**F**: Distal 09 root in contact with mesial root of 10, there was no alveolar bone between the two neighboring teeth in this 61 month old alpaca. **G**/**H**: Deformed distal root of 09 and exposed root canal cavity in a 107 month old llama. Described findings are indicated with white arrows.

The roots of the mandibular molars in general erupted in a vertical manner, with the exception of the distal roots of 09 (in 13 cases = 65%) and 08 (in 8 cases = 40%), which were bent in a caudal direction. This deviation became more prominent with age. In 9 month-old alpaca only a deviation in the distal root of deciduous 08 was observed (Figures 6A, 6B). Later with age, moderate alveolar bone sclerosis around the tooth root obscured the lamina dura and periodontal membrane (Figures 6C, 6D). The neighbouring tooth roots had increasing contact with age and the apex of the distal root of 09 touched the mesial root of 10. A thin layer of bone tissue between the two adjacent tooth roots at the age of about 30 months (Figures 6E, 6F) was seen. Some degree of sclerosis was still present in alveolar bone. At the age of 60 months, when all the teeth were present,^1^ the distal root of the 09 seemed to push the mesial root of the 10 in a caudal direction. At this time no alveolar bone was detected between the two roots (Figures 6G, 6H). In 2 cases (10%) the cementum of the distal root of 09 was missing and the root canal cavity was exposed to the surface of the adjacent tooth root (Figure 6H). No sclerosis was present around the tooth roots at this age, and the periodontal ligament appeared smooth and narrow with soft tissue opacity.

A similar deviation was detected in the distal root of 10′s in 3 cases (15%), but the distance between the distal root of 10′s and the mesial root of 11′s was larger and deformities were less frequent compared to the aforementioned dental roots.

### Maxillary cheek teeth

**Figure 7:**
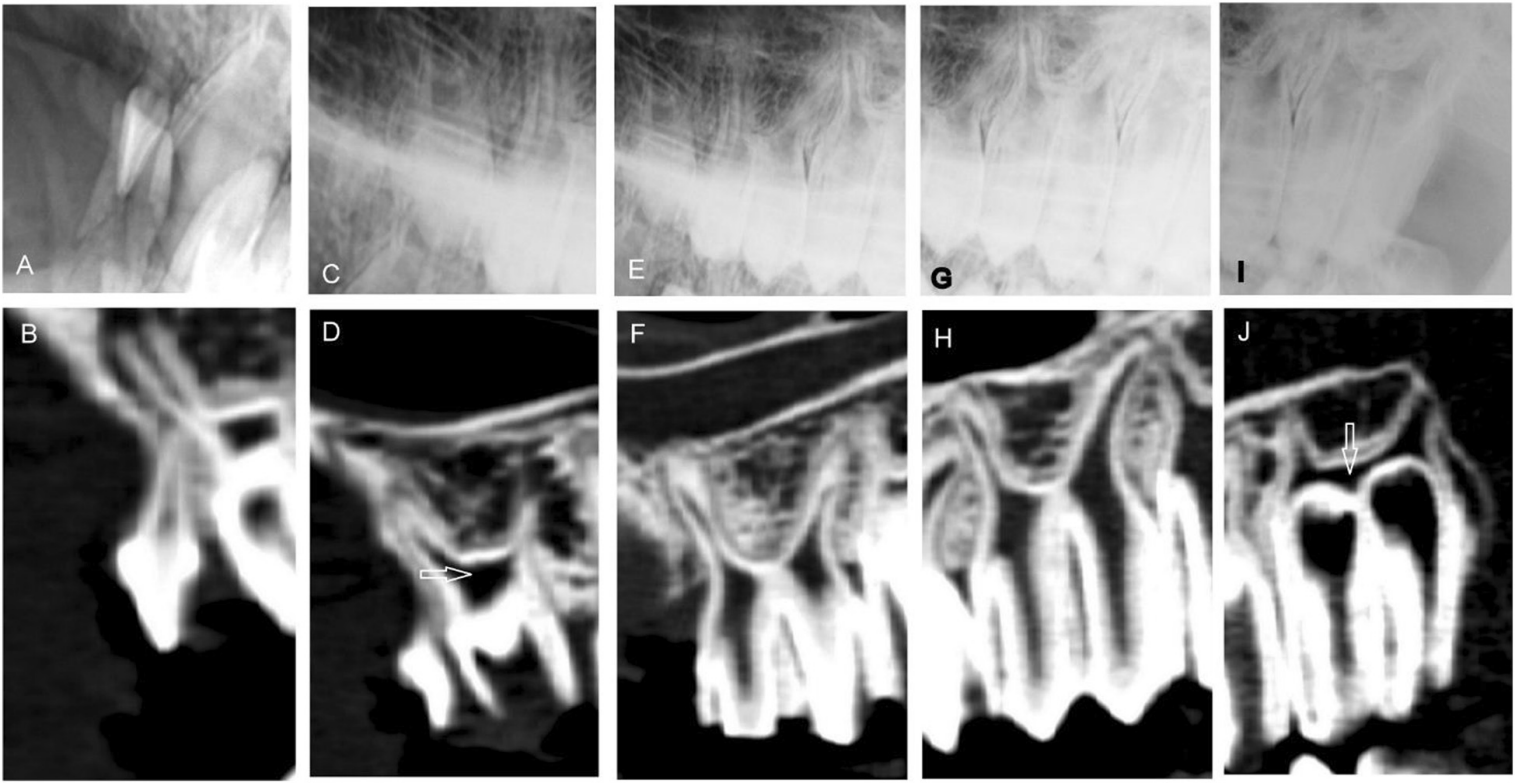
**Maxillary cheek teeth.** Maxillary cheek teeth of an adult llama (age 44 months). There were two premolars and three molars on each side. A/C/E/G/I: Left to right lateral30°dorsal - lateroventral oblique projection showing the right maxilla. B/D/F/H/J: Sagittal CT reconstructions. **A** and **B**: 07, **C** and **D**: 08, **E** and **F**: 09, **G** and **H**: 10, **I** and **J**: 11. Image D and J show a clear and obvious common pulp chamber (white arrow).

**Figure 8:**
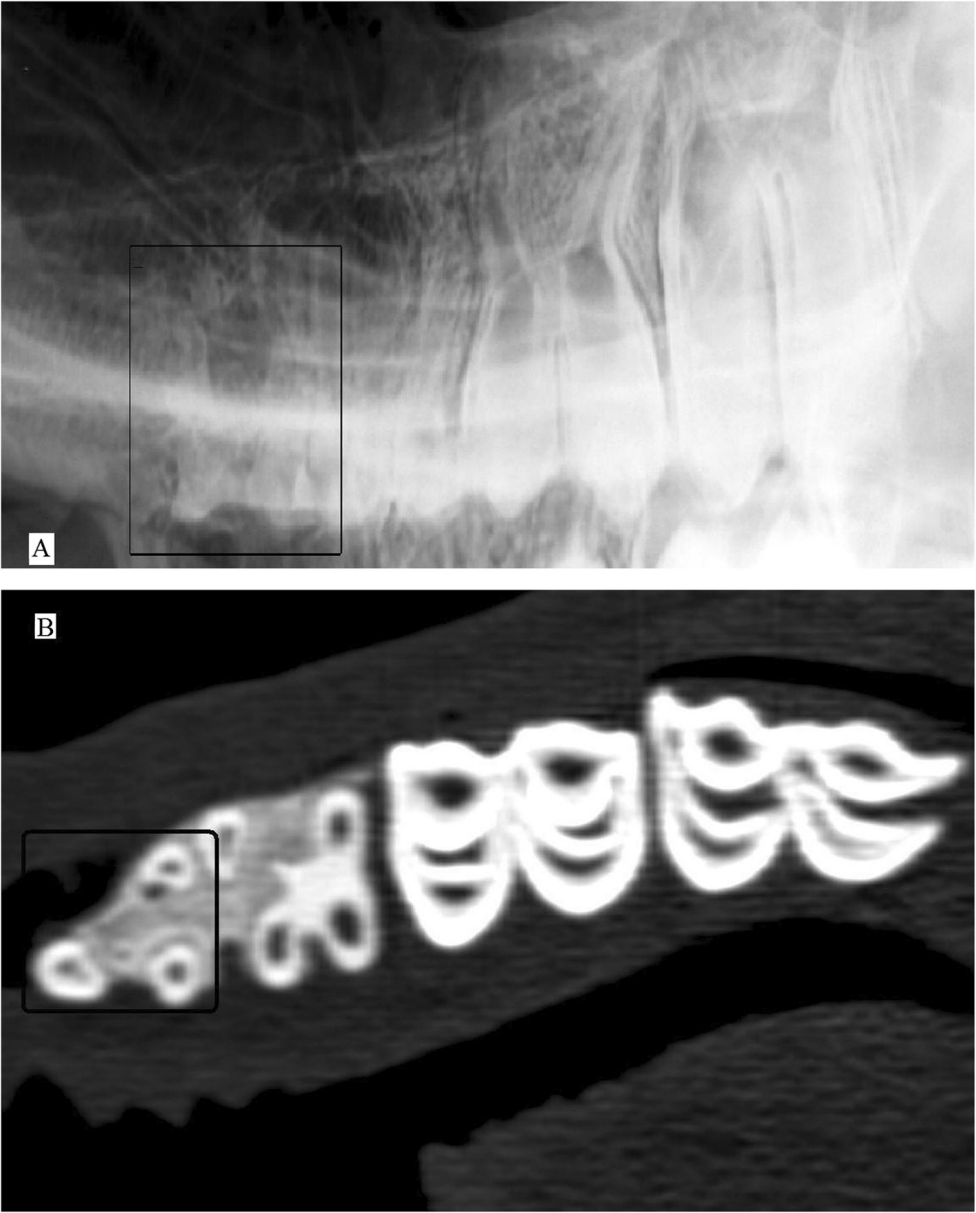
**Divided roots of maxillary 07.** Left to right lateral30°dorsal - lateroventral oblique projection of the upper jaw of a 31 month old male llama **(A)**. The roots of 07 are finally divided into three short horns. This is best visualized in dorsal reconstruction CT images of the maxilla **(B)** where the transverse cross section of the root horns are illustrated as round separate structures in the alveolar bone. Tooth 07 is marked by black box in both images.

South American camelids had five maxillary cheek teeth in each arcade (Figure 7). The third premolar (07) was present in 17 (85%) of the cases and it was the smallest tooth of the arcade. It had a conical shape in sagittal section and lateral oblique view. It contained one pulp horn cavity and no infundibulum. The root was finally divided into three short horns: this was best visualized in MPR reconstructed dorsal plane CT images of maxillary teeth roots (Figure 8).

The fourth premolar (08) had a rectangular shape in sagittal section. It consisted of one column and one or two palatal and two buccal roots. Each root contained one root canal and the two root canals of the buccal roots were fused to one buccal pulp horn. The enamel folding formed one infundibulum in this tooth. The roots of this tooth lay ventrally to the infraorbital foramen (Figures 7C, 7D).

All three maxillary molars varied in size: the 10′s were the largest teeth in this arcade and had rectangular shape and structure. All molars had two columns, four roots and two infundibula. Every column had a palatal and a buccal root, which were superimposed on radiographs. The infundibular funnels expanded toward the distal columns. The distal column of the third molar (11) had the widest infundibular funnel. These funnels were crescent shaped in cross section and located between the palatal and buccal pulp horns, the convex aspect showing to the palatal side.

The maxillary cheek teeth were rectangular in shape (except for 07′s which was oval on cross section) with a lingually located dome-shaped aspect in contrast to the lower jaw in which the vaults were buccal. Although the teeth were located side by side in the same plane, the roots deviated from this plane. They usually got longer with age, but in general there was no deviation toward other roots like in the lower jaw with the exception of 7 cases (35%), where there was contact between the apical end of the disto-buccal root of 09 and the mesio-buccal root of 10 (in analogy to Figures 6E, 6F).

The normal range of the CT radio density of different cheek teeth and different dental tissues is presented in the Tables 4 and 5.

**Table 4:** Radiodensity of permanent cheek teeth

**Tooth no.**	L07*	**L08**	**U07**	**U08**	**L09**	**L10**	**L11**	**U09**	**U10**	**U11**
**Enamel**	2162.1	2673.8	2376.1	2723.8	2738.8	2672.7	2723.1	2688.6	2673.3	2555
**Cementum**	1449.0	1547.4	1312.0	1418.8	1364.2	1284.4	1301.3	1270.9	1198.5	968.3
**Dentine**	1477.9	1481.8	1378.4	1565.8	1435.7	1273.5	1227.2	1312.8	1151.0	953.3
**Pulp**	866	463.2	657.0	208.2	136.3	33.6	7.9	53.7	33.4	-26.7

Mean radiodensity in Hounsfield units of different permanent cheek teeth (*L = Lower, U = Upper).

**Table 5:** Radiodensity of dental tissues in deciduous cheek teeth

**Tooth no.**	L07*	**L08**	**U07**	**U08**
**Enamel**	2380	2452.0	2337.9	2503.6
**Cementum**	1451.9	1333.1	1404.6	1222.1
**Dentine**	1372.7	1310.3	1249.6	1364.7
**Pulp**	891.3	91.8	107.0	84.7

Mean radiodensity in Hounsfield units of different deciduous cheek teeth (*L = Lower, U = Upper).

## Discussion

This study was designed to assess the dental structures of SAC using CT, to compare these findings with radiographic findings and to determine a clinically useful radiographic examination protocol for a thorough dental examination. No data was available on this topic in the literature. CT imaging data in cases of disease and on the normal nondental structures of the skull of SAC do, however, exist [1,2,5,6,9,12]. On the basis of the CT images, the best angulations for oblique radiographic projections can now be recommended and are lateral30°dorsal - lateroventral and lateral30°ventral - laterodorsal respectively for maxillary and mandibular arcades, which provides the least distortion of the reserve crown and tooth roots and the least superimposition of the dental arcades. This result differs from the recommendations of Niehaus where angle of 45° oblique projections was used [2]. In contrast to the study of Niehaus, the goniometer of the X- ray machine was used for determining the incident angle, which seems to be more reliable. As the images in this study have been produced from specimens, Figure 1 show the recommended radiographic technique on live animals based on the actual results.

In the early stages of development of cheek teeth, even with some degree of mineralization in the tooth bud, it was not possible to distinguish the dental sac in the alveolar bone by radiography. However, CT images provided clear visualization of these structures. The dental sacs of 2 to 4 year- old horses have been described [13]. The data of the present study are consistent with those of other studies on horses [13]. As in horses, the buds of the permanent unerupted teeth contained a developing enamel crown. At this stage, these teeth lack tooth root development and have wide pulps and open root apices. After complete eruption, the morphology of the teeth did not change with age and wear of the cheek teeth and enamel overgrowths did not develop as occurred in horses [3,10,14].

Some authors have speculated that premature removal of deciduous caps shortly after eruption can be a potential etiology for dental problems [1,2,6]. As the dental problems in SAC are more frequently seen in mandibular molars [1,2] at the age of 4 to 8 years [2,6] - according to our findings - another possible explanation for this might be that the roots of mandibular cheek teeth were deviated, deformed or shortened and partially destroyed with different degrees of pulp exposure. This fact might explain why the onset of the disease occurs at a wide range of age. These deformities were detected most frequently in mandibular 09′s and with a lower frequency in mandibular 08′s and 10′s, where the dental problems were also most frequently reported to occur [1,2]. In the maxillary arcades, there may be some degree of contact between the roots at the apex. This deviation was commonly detected between the disto-buccal root of the first molar (09) and the mesio-buccal root of second molar (10). Sclerosis of alveolar bone and tooth root deformities were not clearly visualized as in mandibular tooth roots. However, considering the rather small sample size and the lack of animals at specific age categories (median age of the animals was 3 years and 3 months ranging from 9 months to 9 years), caution must be applied, as the findings might not be fully representative for the entire population of SAC.

The fact that the pulp horns in the mesial and distal tooth roots of cheek teeth in SAC do not communicate is reported [1,2]. This statement requires a detailed explanation. As teeth age in healthy SAC, secondary dentine closes the common pulp chamber. The precise moment at which this closure occurs in both healthy and diseased teeth, cannot be determined from our results and requires further investigation. However, we found that a common pulp chamber definitely existed in the younger teeth of SAC. This may be an indicator for surgical intervention.

Concerning the mandibular incisors there was a clear morphological difference between alpacas and llamas as was also published by other authors [3]. Concerning the ongoing eruption process during maturity, further investigations need to be done to understand this better.

There are similarities between the density ranges for dental areas in SAC expressed by Hounsfield Units in this study and those described previously for horses [15,16]. In contrast to the radiopacity in radiographs, the density expressed in Hounsfield units allows an objective comparison between healthy and altered dental tissues in SAC as well as in horses. Furthermore, this value might help to recognize dental tissues for example in aggressive bone lesions of the upper and lower jaw in SAC. The information of CT-findings provides greater detail in the evaluation of dental structures than radiographs. The superimposition of bony and dental structures, especially tooth roots of the maxillary cheek teeth, in radiographic projections does not permit accurate examination, while CT images allow improved identification of the shape and size of different dental structures and their variation and of potential pathologies.

### Consent

Consent was obtained from the owners of the SAC for publication of this study and any accompanying images.

## Conclusions

The present study provides detailed information about the dental imaging morphology in clinically healthy SAC. This provides fundamental knowledge for evaluating images and treatment planning in SAC with dental disease. Future investigations are warranted to test the hypothesis that tooth root deformities, concurrent alveolar sclerosis and interdental tooth root contact might represent predisposing factors for dental disease in SAC.

### Endnotes

^a^Philips Brilliance, Royal Philips Electronics, Netherlands

^b^Siemens Optitop, Siemens Corporation, USA

^c^Fuji FCR 5000, FUJIFILM Corporation, Japan

^d^Fuji IP cassette type CC, FUJIFILM Corporation, Japan

^e^Fuji IP cassette 3A, FUJIFILM Corporation, Japan

^f^efilm 3.0.1

^g^Excel 2007, Microsoft Incorporation

## Competing interests

The authors declare that they have no competing interests.

## Authors’ contributions

PZ initiated, planned and supervised the study. AR carried out the radiographic and CT examinations under supervision of UG. FS advised AR in dental nomenclature. All authors have read and approved the final manuscript.
